# EPIGENETICS MARKERS OF AGING AND EXPOSURES

**DOI:** 10.1093/geroni/igz038.2695

**Published:** 2019-11-08

**Authors:** Joanne Murabito

**Affiliations:** Boston University, Boston, Massachusetts, United States

## Abstract

Widespread changes to the epigenome occur with aging. DNA methylation is one of the most commonly studied epigenetic mechanisms, reflects lifetime exposures that impact aging, and is associated with age-related disease risk. Many longitudinal cohort studies have existing cross-sectional or longitudinal DNA methylation data along with genotype and expression data permitting investigation of relationships between DNA methylation markers, exposures, and disease. The data can be leveraged to conduct large epigenome-wide association studies (EWAS) of aging and age-related disease to identify DNA methylation biomarkers and lead to insights into novel biologic pathways for development of interventions to delay aging. DNA methylation age measures robustly predict chronologic age and associate with both healthspan and lifespan. During the workshop, examples from cohort studies and the CHARGE consortium will be presented.

